# Performance of a Deep Learning System and Performance of Optometrists for the Detection of Glaucomatous Optic Neuropathy Using Colour Retinal Photographs

**DOI:** 10.3390/bioengineering11111139

**Published:** 2024-11-13

**Authors:** Catherine L. Jan, Algis Vingrys, Jacqueline Henwood, Xianwen Shang, Christian Davey, Peter van Wijngaarden, George Y. X. Kong, Jennifer C. Fan Gaskin, Bernardo P. Soares Bezerra, Randall S. Stafford, Mingguang He

**Affiliations:** 1 Centre for Eye Research Australia, Royal Victorian Eye and Ear Hospital, East Melbourne, VIC 3002, Australia; 2 Ophthalmology, Department of Surgery, The University of Melbourne, Melbourne, VIC 3010, Australia; 3 Lost Child’s Vision Project, Sydney, NSW 2000, Australia; 4 Department of Optometry and Vision Sciences, The University of Melbourne, Melbourne, VIC 3053, Australia; 5 School of Mathematics and Statistics, University of Melbourne, Melbourne, VIC 3010, Australia; 6 Stanford Prevention Research Center, Stanford University School of Medicine, Stanford, CA 94304, USA; 7 School of Optometry, The Hong Kong Polytechnic University, Kowloon, Hong Kong; 8 Research Centre for SHARP Vision (RCSV), The Hong Kong Polytechnic University, Kowloon, Hong Kong; 9 Centre for Eye and Vision Research (CEVR), 17W Hong Kong Science Park, Hong Kong

**Keywords:** artificial intelligence, glaucoma detection, primary care, deep learning

## Abstract

Background/Objectives: Glaucoma is the leading cause of irreversible blindness, with a significant proportion of cases remaining undiagnosed globally. The interpretation of optic disc and retinal nerve fibre layer images poses challenges for optometrists and ophthalmologists, often leading to misdiagnosis. AI has the potential to improve diagnosis. This study aims to validate an AI system (a convolutional neural network based on the Inception-v3 architecture) for detecting glaucomatous optic neuropathy (GON) using colour fundus photographs from a UK population and to compare its performance against Australian optometrists. Methods: A retrospective external validation study was conducted, comparing AI’s performance with that of 11 AHPRA-registered optometrists in Australia on colour retinal photographs, evaluated against a reference (gold) standard established by a panel of glaucoma specialists. Statistical analyses were performed using sensitivity, specificity, and area under the receiver operating characteristic curve (AUROC). Results: For referable GON, the sensitivity of the AI (33.3% [95%CI: 32.4–34.3) was significantly lower than that of optometrists (65.1% [95%CI: 64.1–66.0]), *p* < 0.0001, although with significantly higher specificity (AI: 97.4% [95%CI: 97.0–97.7]; optometrists: 85.5% [95%CI: 84.8–86.2], *p* < 0.0001). The optometrists demonstrated significantly higher AUROC (0.753 [95%CI: 0.744–0.762]) compared to AI (0.654 [95%CI: 0.645–0.662], *p* < 0.0001). Conclusion: The AI system exhibited lower performance than optometrists in detecting referable glaucoma. Our findings suggest that while AI can serve as a screening tool, both AI and optometrists have suboptimal performance for the nuanced diagnosis of glaucoma using fundus photographs alone. Enhanced training with diverse populations for AI is essential for improving GON detection and addressing the significant challenge of undiagnosed cases.

## 1. Introduction

Glaucoma is the leading cause of irreversible blindness [1,2], and its prevalence is expected to increase as the population ages [3]. This progressive disease damages the optic nerve, causing irreversible vision loss if left untreated [4]. Glaucoma is often asymptomatic in its early stages, making regular eye exams crucial for early detection and treatment [5]. Despite increasing awareness, the fraction of glaucoma cases that remain undiagnosed is as high as over 60% in Australia and over 70% globally [6,7].

One of the challenges in the management of glaucoma is the interpretation of optic disc and retinal nerve fibre layer (RNFL) appearance. This task requires specialised training and skills and can be time-consuming. In addition, there can be significant variability in the interpretation of optic nerves and RNFL by different clinicians, leading to potential errors and misdiagnosis [8]. In many countries such as Australia, the USA, the UK, New Zealand, and Canada, optometrists are primary healthcare professionals responsible for the diagnosis and management of glaucoma. However, the performance of optometrists and ophthalmologists in glaucoma detection is suboptimal [9]. An Australian study reported that out of a group of undiagnosed glaucoma cases with a cup-to-disc ratio (CDR) > 0.7, 65% had seen an optometrist and 48% had seen an ophthalmologist in the past year [6]. A more recent study from Sydney audited the appropriateness of glaucoma care delivery from a nationally representative sample of 42 optometry clinics: it showed that most optometrists performed appropriate assessments in terms of evaluating the optic nerve head, such as recording the cup-to-disc ratio, a biomarker of the disease, (93%), the pattern of the neuroretinal rim (46%), and the size of optic disc (6%), as well as obtaining fundus images (78%) [10]. However, “appropriate care” was defined simply as documentation of the clinical assessment in the patient files and does not reflect the accuracy of the assessment. There is currently no study that reports the accuracy of glaucoma detection by Australian optometrists.

Artificial intelligence (AI) algorithms have the potential to bridge the gap in healthcare access and to reduce inter-observer variation in the interpretation of images for the detection of glaucomatous optic neuropathy (GON). AI systems use machine learning algorithms to analyse large datasets of images and learn patterns and features that are characteristic of the condition being considered. AI algorithms can analyse fundus images rapidly, reducing the demand for manpower and allowing for the early detection of GON. This is especially important in areas where there is a shortage of trained eye professionals and a high demand for disease detection.

An AI system for the classification of GON based on colour fundus photographs has been developed using a Chinese population [11]. Despite its high performance (an AUC of 0.986 with a sensitivity of 95.6% and specificity of 92.0%), a major limitation of this system, and deep learning systems in general, is that it was developed using one ethnic group [11]. This may result in reduced performance when applied to populations distinct from those on which they were trained. This limitation underscores the need for validation of AI model performance with different racial and ethnic subgroups before AI systems can be translated into global applications. To our knowledge, there has been no study that externally validated an AI algorithm for glaucoma detection using an ethnically different population, marking our study the first of its kind to do so.

This study aims to (1) validate an AI system developed using retinal images from Chinese participants using an ethically different population (the UK Biobank) and (2) compare the AI system with Australian optometrists in the performance of GON classification.

## 2. Methods

We performed a retrospective, external validation study comparing the performance of an AI algorithm against Australian Health Practitioner Regulation Agency (AHPRA)-registered optometrists in Australia for detecting GON from colour retinal photographs.

Ethics approval was granted by the Human Research Ethics Committees of St. Vincent’s Hospital, Melbourne, Australia (Project ID 86246, St Vincent’s HREC Ref: HREC 122/22), and the study was conducted in accordance with the Declaration of Helsinki. All clinician participants provided online informed consent. The approving ethics committee waived the requirement for informed consent from patients from whom the fundus photos were taken due to retrospective nature of the study and the use of fully anonymised fundus photographs. This study follows the Standards for Reporting of Diagnostic Accuracy (STARD) reporting guideline [12].

### 2.1. Algorithm Overview

The algorithm used for our study is described in detail by Li et al. [2] In brief, a deep neural network was initially trained in 2016, incorporating the ISGEO definition for glaucoma alongside classical glaucoma signs such as disc haemorrhage and retinal nerve fibre layer (RNFL) defects (refer to Table 1 [11]). Notably, only structural features identified in monoscopic fundus photographs were considered for analysis. The algorithm comprised a convolutional neural network based on the Inception-v3 architecture and was trained to classify digital colour retinal photographs into 4 categories of GON grading: unlikely, suspect, certain, and ungradable [11]. The image pixel values were scaled to values in a range of 0 through 1 and then downsized to a 299 × 299 matrix. Using the ground truth grade based on consensus gradings of glaucoma specialists, 2 levels of detection of referable GON were adopted for this deep learning system: non-referable GON and referable GON (consisting of suspected and certain GON) [11].

With ophthalmologists’ grading process, each retinal photograph was graded between 3 and 9 times. Among 39,745 fully gradable retinal photographs, 8000 images were selected randomly using simple random sampling and treated as the validation set, and the remaining 31,745 images were used as the training set [11]. Under controlled laboratory conditions, this AI model demonstrated an area under the receiver operating characteristic curve (AUROC) of 0.986 (95% confidence interval, 0.985–0.988) for referable GON. An accuracy of 92.9% with a sensitivity of 95.6% and a specificity of 92.0% was obtained for the classification of referable GON [11].

### 2.2. Study Population

The UK Biobank recruited half a million people aged 40–69 years in 2006–2010 from the UK [13]. Participants underwent physical assessments, biospecimen collection, provided detailed information about themselves and agreed to have their health followed. A subset of 68,544 participants underwent retinal fundus imaging. Images were non-mydriatic, single field colour fundus photographs (45-degree field of view, centred to include both optic disc and macula) captured using a digital Topcon-1000 integrated ophthalmic camera (Topcon 3D OCT1000 Mark II, Topcon Corp., Tokyo, Japan). Images were stored in PNG format with dimensions 2048 × 1536 pixels [13]. The UK Biobank obtained approval for data collection from the Northwest Region NHS research ethics committee. Approval for access to UK Biobank data for the purpose of this study was granted under agreement between the UK Biobank and the Centre for Eye Research Australia.

### 2.3. Grading and Adjudication

**Figure 1:**
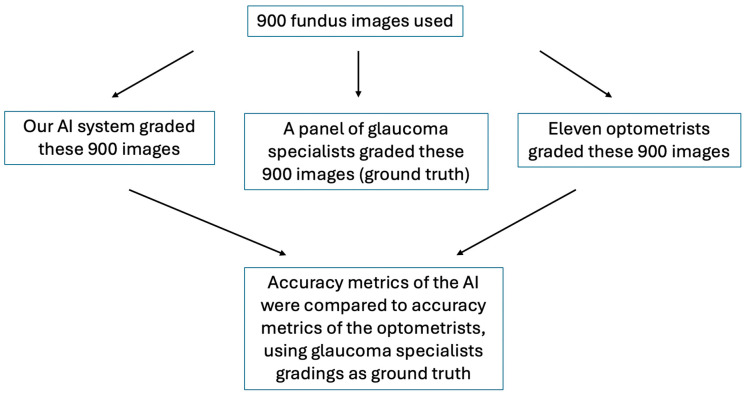
Flow diagram of research process.

The AI algorithm classification for UK Biobank retinal images was compared against the classification by 11 Australian board-certified optometrists. Nine hundred images were selected (see detail below under sample size calculation) that were deemed gradable for GON by both the AI and an optometrist on the research team, and then the participant optometrists assessed the likelihood of GON, defined as certain, suspect, or unlikely (Figure 1) [11]. Graders were blinded to the AI and the reference standard (ground truth), and no additional clinical information was provided.

The reference standard (ground truth) consisted of a consensus grade (unlikely, suspect, or certain, Table 1) applied to each image by a panel of two Australian board-certified, fellowship-training glaucoma specialists (Figure 1). In cases of discrepancy, the images were adjudicated by a third glaucoma specialist. For “glaucoma certain” and “glaucoma suspect” cases, the specialists further noted down the following features influencing their decision: large CDR, localised thinning of neural rim (notching), presence of disc haemorrhages, presence of PPA in non-myopic eye, RNFL defects, and/or other.

### 2.4. Outcome Measures

The primary outcome was the accuracy metrics (sensitivity, specificity, AUROC, accuracy, and normalised Matthews correlation coefficient (nMCC)) of GON classification by the AI and the optometrists, consisting of referable (certain plus suspect) or non-referable (unlikely), compared to the gold standard grading results provided by the glaucoma specialists. The secondary outcome was the accuracy metrics of GON classification by the AI and the optometrists, consisting of glaucoma certain compared to the gold standard grading results provided by the glaucoma specialists. A further secondary outcome included the reason for false negative cases from AI and optometrist classifications for identifying ‘referable glaucoma’ and ‘glaucoma certain’ cases when compared with specialist diagnosis.

### 2.5. Statistical Analysis

All study data were manually entered and managed using REDCap electronic data capture tools (REDCap or Research Electronic Data Capture, Vanderbilt University, Nashville, TN, USA) which were hosted at the Centre for Eye Research Australia (CERA). De-identified data were downloaded from REDCap and imported into Stata/IC version 17 (College Station, TX, USA) for statistical analysis. Two-by-two tables were generated to characterise the sensitivity and specificity of the AI system and optometrists (index tests) with respect to two-ophthalmologist adjudication (ground truth), at the image level. McNemar’s test was used to compare the sensitivity and specificity of AI with those of optometrists. The roccomp command in Stata was used to compare the equality of the areas under two AUROCs.

### 2.6. Sample Size Calculation

The sensitivity of the algorithm was assumed to be 95.6%, the specificity was assumed to be 92.0% [11] and the prevalence of referable glaucoma was 5.25% according to the AI grading. Calculations were performed in the R statistical computing language version 2022, using Wilson confidence intervals for the binomial parameters. Under these assumptions, the positive and negative predictive values (PPVs and NPVs) were determined to be 0.305 and 0.998, respectively. A maximum number of 900 images could be feasibly reviewed by participating optometrists, so the PPV and NPV were used to determine how many of these images needed to be positively classified by the AI and how many needed to be negatively classified. This resulted in the required numbers of 114 positive (glaucoma certain plus glaucoma suspects) and 786 negative AI-classified images. We have chosen 900 images accordingly (Figure 1).

## 3. Results

Eleven eligible optometrists participated in the grading task: four (36.4%) were male and seven (63.6%) were female. Years of practising optometry ranged from 3 to 42 years with mean of 14 years and median of 12 years. Ten (90.9%) were therapeutically qualified (have a licence to prescribe ophthalmic medication including anti-glaucoma eye drops). Two (18.2%) work in a public-sector optometry practice and nine (81.8%) in private-sector practises. Five (45.5%) work in a corporate setting, five (45.5%) as independents, and one (9.1%) at an educational facility. Seven (63.6%) work full-time (practising 16 h or more per week) and four (36%) work part-time (less than 16 h per week).

### 3.1. Sensitivity, Specificity, AUROC, and Accuracy of AI Compared to Glaucoma Specialists Versus Those of Optometrists Compared to Glaucoma Specialists

Table 2 summarises the sensitivity, specificity, and AUROC of AI and optometrists compared to glaucoma specialists. For referable GON, the sensitivity of the AI (33.3% [95%CI: 32.4–34.3]) was significantly lower than that of optometrists (65.1% [95%CI: 64.1–66.0]), *p* < 0.0001; however, the specificity was significantly higher (AI: 97.4% [95%CI: 97.0–97.7]; optometrists: 85.5% [95%CI: 84.8–86.2], *p* < 0.0001). The AUROC was significantly higher for optometrists (0.753 [95%CI: 0.744–0.762]) than for the AI system (0.654 [95%CI: 0.645–0.662], *p* < 0.0001). For the detection of ‘certain’ GON, the AI system achieved a sensitivity of 28.6% (95%CI: 27.7–29.5), which was similar to that of optometrists (28.0%; 95%CI: 27.1–28.9; *p* = 0.9); the specificity for this classification task was 94.6% (95%CI: 94.2–95.0), 95.2% (95%CI: 94.8–95.6; *p* = 0.1), respectively, with an AUROC of 0.616 (95%CI: 0.597–0.635) for both AI and the optometrists, *p* = 1.0.

Furthermore, the AI system achieved a true negative rate of 0.97, a true positive rate of 0.33, a false positive rate of 0.03, a normalised Matthews correlation coefficient (nMCC) of 0.72, and an accuracy of 0.76 in diagnosing referable glaucoma cases, compared to grading by glaucoma specialists. Optometrists achieved a true negative rate of 0.98, a true positive rate of 0.33, a false positive rate of 0.02, an nMCC of 0.73, and an accuracy of 0.78. These results suggest that the AI system performs well. Its diagnostic accuracy and ability to correctly identify non-diseased cases are comparable to those of optometrists. The slightly higher true negative rate and lower false positive rate of optometrists indicate a marginally better performance in distinguishing between healthy and referable glaucoma cases. However, both demonstrate similar true positive rates, highlighting their comparable effectiveness in detecting referable glaucoma when it is present. Overall, these findings suggest that while the AI system is a promising tool, optometrists currently offer slightly superior diagnostic performance.

### 3.2. False Positive and False Negative Cases

Each of the 900 retinal images were graded by 11 optometrists, yielding a total of 9900 image gradings. For referable GON detection by AI, the number of false positive cases was 176 (false positive rate 2.64%) and the number of false negative cases was 2156 (false negative rate 66.7%) (Table 3). For referral GON detection by optometrists, the number of false positive cases was 966 (false positive rate 14.5%) and the number of false negative cases was 1130 (false negative rate 34.9%) (Table 4). For GON certain detection by AI, the number of false positive cases was 506 (false positive rate 5.41%) and the number of false negative cases was 385 (false negative rate 71.43%). For GON certain detection by optometrists, the number of false positive cases was 452 (false positive rate 4.83%) and the number of false negative cases was 388 (false negative rate 72.0%).

### 3.3. Reasons for False Negative Cases

**Figure 2:**
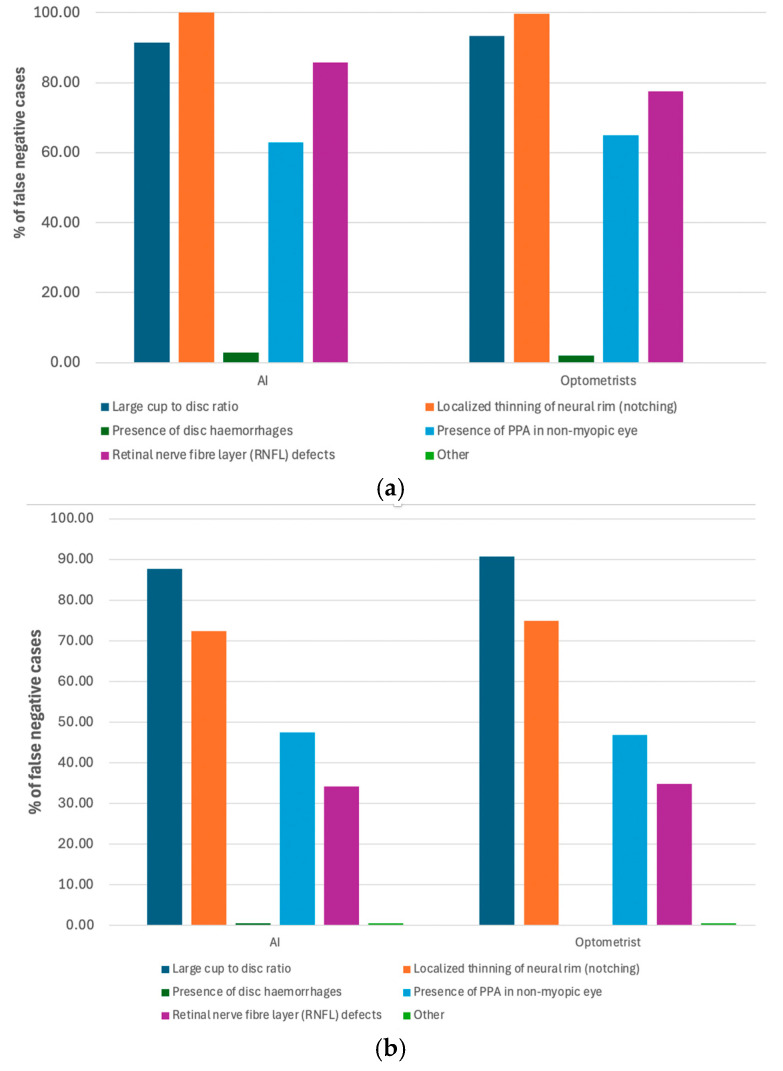
(**a**) Features in false negative cases from AI and optometrist classifications for identifying ‘glaucoma certain’ cases when compared with specialist diagnosis. (**b**) Features in false negative cases from AI and optometrists’ classifications for identifying referral cases (certain plus suspect) when compared with specialist diagnosis.

Figure 2a,b demonstrate the reasons for glaucoma specialists’ diagnoses in the case of false negative categorisation. For ‘referable glaucoma’ cases (certain plus suspect), the top three reasons were large cup-to-disc ratio, localised thinning of neural rim (notching), and presence of PPA in non-myopic eyes, from both AI and optometrists’ gradings compared to glaucoma specialist ophthalmologists’ grading. For ‘glaucoma certain’ cases, the top 3 reasons for false negative diagnosis were localised thinning of neural rim (notching), large cup-to-disc ratio, and RNFL defects, from both AI and optometrists’ gradings compared to glaucoma specialist ophthalmologists’ grading.

Note that there were 233 (26%) disagreements between the first glaucoma specialist and the second glaucoma specialist, suggesting that detecting glaucoma using fundus images alone can produce highly variable results, even by highly trained specialists.

### 3.4. Sensitivity, Specificity, and AUROC of AI Compared to Optometrists

We also compared AI directly to the optometrists without using glaucoma specialist grading (ground truth) to see how similar their performances were. For detecting referable GON, the AI system demonstrated a sensitivity of 81.2% (95%CI: 80.4–82.0%) and specificity of 76.3% (95%CI: 75.4–77.1%), with an AUROC of 0.649 (95%CI: 0.640–0.657), when compared directly to the optometrists. The AI achieved a true negative rate of 0.86, a true positive rate of 0.65, a false positive rate of 0.15, an nMCC of 0.76, and an accuracy of 0.79 when compared with optometrists.

The sensitivity of the AI system for referable glaucoma in this cohort was 33.3%, significantly lower than the expected sensitivity of 95.6%. To assess the adequacy of the study’s power, we calculated the achieved power using a sensitivity of 33.3%, a specificity of 97.4%, a prevalence of referable glaucoma of 15.4% (comprising both glaucoma certain and glaucoma suspect) [14], and a sample size of 900 images graded by 11 optometrists. The predicted power was found to exceed 99.9%, indicating that the study possesses sufficient power to detect the specified outcomes.

## 4. Discussion

Our results demonstrate that the AI system was able to identify GON with a performance similar to optometrists in a cohort of people from the UK. The sensitivity of both AI and optometrists was low for the detection of ‘certain’ GON. For the detection of referable GON, the sensitivity of AI was significantly lower than that of the optometrists but with significantly higher specificity. These results suggest that the reading of colour fundus photos by either AI or optometrists is more suitable as a screening tool for glaucoma, rather than as a diagnostic tool. More detailed analyses, such as from visual field testing, optical coherence tomography, intra-ocular pressure, and family history, are needed to form a clinical diagnosis [9].

This AI algorithm was developed using colour fundus photos from a population of Chinese people. The sensitivity (33.3%) and specificity (97.4%) of referable GON from this external validation set are different from those (sensitivity = 95.6%, specificity = 92.0%) obtained from the original population [11]. The discrepancies may be partially due to variations in ocular anatomy between different ethnicities. Additional ocular and systemic comorbidities may also adversely impact algorithm performance. Further algorithm training with the UK population may improve algorithm performance in this population. Our results highlight the importance of training and validation of AI algorithms using diverse populations to confirm their global applicability and robustness in glaucoma screening and diagnosis. Furthermore, the high specificity and low false positive rate observed in this study suggest that the AI algorithm has the potential to reduce the burden on tertiary hospitals and ophthalmologists by minimising unnecessary referrals of false positive cases. This can lead to more efficient use of healthcare resources and improved patient care by directing specialist attention to cases that are truly at risk for glaucoma or in need of further evaluation. However, the low sensitivity and missed diagnoses is a concern.

There has been no study comparing AI performance in glaucoma detection across different ethnic groups. However, a review of the literature on other eye diseases and systemic conditions reveals that while some AI algorithms perform similarly across various ethnic groups [15], it is not uncommon for these algorithms to demonstrate differing accuracy levels based on ethnicity [16] or even geographic location within the same country [17]. For instance, one study [17] found that an AI algorithm for detecting diabetic retinopathy exhibited varying performance in Seattle compared to Atlanta: the algorithm achieved higher negative predictive values (NPVs) with the Atlanta dataset (ranging from 90.71% to 98.05%) compared to the Seattle dataset (77.57% to 90.66%). Conversely, the positive predictive values (PPVs) in the Atlanta dataset ranged from 24.80% to 39.07%, which were lower than those in the Seattle dataset (42.04% to 62.92%) [17]. This suggests that incorporating racial or ethnic information is crucial for the development and training of AI algorithms. However, reviews indicate that only 1.4% (12 out of 831) of studies reported ethnicity, and just 7.3% (61 out of 831) reported race [18].

The findings from our study reveal significant insights into the diagnostic challenges encountered by AI systems and optometrists compared to glaucoma specialist ophthalmologists in identifying glaucoma, particularly in distinguishing between ‘glaucoma certain’ and ‘referable glaucoma’ cases. In both categories, we identified common reasons for false negative diagnoses across AI and optometrists. Notably, localised thinning of the neural rim (notching), large CDR, and RNFL defects were overlooked. This suggests a pattern where these features, critical in identifying early signs of glaucoma, were potentially overlooked or underestimated in assessments by our AI system and optometrists compared to the assessments made by glaucoma specialist ophthalmologists.

Our study underscores the challenges in achieving diagnostic accuracy in different ethnical groups and using geographically different glaucoma specialists for ground truth. While AI systems have shown promise in automating glaucoma screening, they exhibited similar patterns of false negatives to optometrists. Understanding these specific features contributing to false negative diagnoses is crucial for refining AI algorithms and enhancing the training of non-specialist clinicians. Strategies to improve sensitivity, such as diversifying training data and implementing decision-support tools, could help mitigate diagnostic gaps, necessitating the ongoing need for updating AI algorithms to better align with the diagnostic acumen of specialist ophthalmologists. Our findings demonstrate that AI algorithms exhibit comparable accuracy to licenced optometrists in Australia for detecting referable glaucoma cases. This underscores the potential of AI as a screening tool for glaucoma where there is a shortage of optometrists and ophthalmologists, rather than a clinical tool to be used for glaucoma diagnosis.

However, both the AI and optometrists exhibit limited performance in detecting GON cases using colour fundus photos alone, highlighting the complexity of glaucoma diagnosis and the need for sophisticated clinical care. It would be interesting to see the combined performance of AI with optometrists versus AI or optometrists alone, as this will indicate the potential of AI as a clinical assistance tool for GON detection by optometrists. Our results underscore the potential of AI to support and augment clinical decision-making in glaucoma diagnosis. Integrating AI with optometric practice could enhance diagnostic efficiency and accuracy, particularly if the AI system is continually refined and validated against real-world clinical data. Additionally, exploring how AI can complement optometric evaluations rather than replace them may offer a more balanced approach to leveraging technology in clinical practice.

Our study has several strengths. First, we implemented a consistent and rigorous reference standard to all images for external validation. Second, we presented a variety of composite outcomes (GON unlikely, GON suspect, GON certain) that are clinically relevant to real-world screening programmes. Lastly, our image dataset represents a diverse population from across the UK, enabling generalisation to similar populations in the UK or other countries with comparable demographics, such as Australia. Our study also has several limitations. Despite the use of a rigorous reference standard, we did not assess functional changes associated with glaucoma such as those from visual field testing, or other structural changes such as those from optical coherence tomography. Secondly, ideally, we would want patient data from a local Australian population; however, due to practical and ethical challenges of gaining such data, we used data from the UK Biobank. While this dataset predominantly represents an Anglo-Saxon population, it still offers a closer demographic match to a significant portion of Australia’s population.

Addressing the high prevalence of undiagnosed glaucoma remains a critical priority for Australia and globally, necessitating more research like this into the development and integration of innovative technologies to tackle this pressing issue.

## Figures and Tables

**Table 1:** Classification for GON used to train the AI algorithm being deployed in this study.

Classification	Presence of Clinical Features
Unlikely	Does not meet any of the following criteria
Suspect	Any of the following: 0.7 ≤ VCDR < 0.9 0.05 DD < rim width ≤ 0.1 DD RNFL defect Disc haemorrhage
Certain	Any of the following: VCDR ≥ 0.9 Rim width ≤ 0.05 DD or localised notches RNFL defect corresponds to narrowing of rim or localised notches

DD = disc diameter; RNFL = retinal nerve fibre layer; VCDR = vertical cup-to-disc ratio. Table adopted from Li et al. [11].

**Table 2:** Comparison of AI against 11 optometrists for glaucoma detection in 900 images, with reference to a panel of glaucoma specialists as the gold standard.

	Percent (95% Confidence Interval)		
	AI	Optometrists	*p* Value ^a^
GON certain			
Sensitivity	28.6 (27.7–29.5)	28.0 (27.1–28.9)	0.9
Specificity	94.6 (94.2–95.0)	95.2 (94.8–95.6)	0.1
AUROC	0.616 (0.597–0.635)	0.616 (0.597–0.635)	1.0
Referable GON (GON certain + GON suspect)			
Sensitivity	33.3 (32.4–34.3)	65.1 (64.1–66.0)	<0.0001
Specificity	97.4 (97.0–97.7)	85.5 (84.8–86.2)	<0.0001
AUROC	0.654 (0.645–0.662)	0.753 (0.744–0.762)	<0.0001

^a^ The McNemar test was used to compare the sensitivity and specificity of AI to those of optometrists. The roccomp command in Stata was used to compare the equality of the areas under two receiver operating characteristic curves (AUROCs).

**Table 3:** Confusion matrix for AI grading compared to glaucoma specialist grading (gold standard).

	Specialist Refer	Specialist Non-Refer	Total
AI Refer	1078	176	1254
AI Non-refer	2156	6490	8646
Total	3234	6666	9900

**Table 4:** Confusion matrix for optometrists’ grading compared to glaucoma specialist grading (gold standard).

	Specialist Refer	Specialist Non-Refer	Total
Optometrist Refer	2104	966	3070
Optometrist Non-refer	1130	5700	6830
Total	3234	6666	9900

## Data Availability

Data cannot be shared publicly due to privacy protection of the participants and ethical restrictions. For researchers interested in the data, requests can be made to the corresponding authors jancj@student.unimelb.edu.au or mingguang.he@unimelb.edu.au.
